# *Haemonchus contortus* Infection Alters Gastrointestinal Microbial Community Composition, Protein Digestion and Amino Acid Allocations in Lambs

**DOI:** 10.3389/fmicb.2021.797746

**Published:** 2022-02-11

**Authors:** Hai Xiang, Yi Fang, Zhiliang Tan, Rongzhen Zhong

**Affiliations:** ^1^Jilin Provincial Key Laboratory of Grassland Farming, Northeast Institute of Geography and Agroecology, Chinese Academy of Sciences, Changchun, China; ^2^College of Advanced Agricultural Science, University of Chinese Academy of Sciences, Beijing, China; ^3^CAS Key Laboratory for Agro-Ecological Processes in Subtropical Region, Institute of Subtropical Agriculture, Chinese Academy of Sciences, Changsha, China

**Keywords:** digestion, growth performance, gastrointestinal microbiota, *Haemonchus contortus*, protein metabolism, sheep

## Abstract

The objective of this study was to investigate associations between gastrointestinal microbiota and protein metabolism of lambs infected with *Haemonchus contortus*. Sixteen male Ujumqin lambs (initial body weight = 32.4 ± 3.9 kg) were dewormed and randomly assigned to 2 equal groups, to be infected or not infected with *Haemonchus contortus* (GIN and CON, respectively). The experiment lasted 77 days. The GIN lambs had lower packed cell volume (PCV) and increased wormegg count (WEC) after 14 days. Furthermore, in infected lambs, there were decreases in apparent digestibility of dry matter (*P* = 0.011), crude protein (*P* = 0.004) and ether extract (*P* = 0.007), as well as decreased ruminal pepsin (*P* < 0.001) and lipase (*P* = 0.032) activity but increased ruminal α-amylase (*P* = 0.004) and cellulase activity (*P* = 0.002), and decreased jejunal α-amylase activity (*P* = 0.033). In addition, infection with *H. contortus* decreased alpha diversity of the gastrointestinal microbial community in the rumen, abomasum and duodenum, although microbiota associated with carbohydrate and proteolytic metabolism were increased and up to 32 KEGG pathways in the duodenum were predicted to be significantly affected. In conclusion, *H. contortus* infection in lambs altered the gastrointestinal microbial community composition and disturbed protein digestion and allocation of absorbed amino acids. These results provided insights into consequences of *H. contortus* infection in lambs and could facilitate development of novel nutritional strategies to improve animal health.

## Introduction

Gastrointestinal nematodes (GINs) have been confirmed to be an important constraint to efficient ruminant production of worldwide (Wang et al., 2017). In particular, the nematode *Haemonchus contortus* of Trichostrongylidae family, a voracious abomasal blood feeder, has many deleterious effects on ruminants, including blood loss and inducing life-threatening anemia, which represent the primary constraint to profitable sheep and goat production in many regions of the world (Albers et al., 1990). It is well known that parasitism and malnutrition often occur concurrently, especially with inadequate dietary protein (amino acids). Altered protein metabolism in ruminants infected with GINs has been reported (Coop and Holmes, 1996). Furthermore, GIN infections decreased dietary protein utilization associated with tissue deposition, bone growth and wool production of sheep (Coop and Kyriazakis, 1999; Yu et al., 2000) but increased protein metabolism associated with tissue repair and immune responses (Ramanan et al., 2016). Furthermore, individual amino acids are also disturbed by GIN infections in sheep. For instance, the small intestine’s irreversible loss rate of methionine (Met) and cysteine (Cys) decreased in sheep infected with *Trichostrongylus colubriformis*, although the whole-body irreversible loss rate of Cys and valine (Val) was not affected (Bermingham, 2000). In addition, effects of GIN infections on host amino acid metabolism varied with the duration of infection (Yu et al., 2000). Mechanisms of altered dietary protein metabolism in lambs infected with *H. contortus* remain unclear, but may be related to the host’s gastrointestinal microbiota (Dai, 2011; Wu et al., 2020).

Countless microbes, including bacteria, archaea, fungi, protozoa, and viruses inhabit the gastrointestinal tract of ruminants and constitute the gut microbiota. This complex microbial community has an important role in ruminant physiology, including nutrient metabolism, immune system development and defense against pathogens (Peachey et al., 2017). Furthermore, the gastrointestinal tract is also home to various species of GINs (Wang et al., 2017). Parasitic GINs not only decrease host ruminant productivity, but also significantly alter the structure and function of their symbiotic gastrointestinal microbiome. Briefly, GIN infection increases the bacterial load but decreases the abundance of archaea in the abomasum and changes the function of microbiota, such as immunological pathways, energy homeostasis and nutrition metabolism (Li et al., 2016; Allison et al., 2018). Furthermore, these effects on gastrointestinal microbiota vary with duration of infection (Cortés et al., 2020). Increasing evidence, particularly in human and rodent models of helminth infection, indicate a multitude of interactions between parasites and gut microbiota, with profound impacts on host immunity and nutritional metabolic potential (Peachey et al., 2017). However, exploration of the relationship between the changes of gastrointestinal microbiota and the effects of nutritional metabolism in ruminants infected by GINs is fragmentary and not systematic, so, further research is needed.

The objective was to investigate effects of GIN infection on host lambs’ protein digestion and amino acid profiles, as well as gastrointestinal microbiota composition, biological pathways, and functional categories, to provide insights into how GIN affect gastrointestinal microbiota and protein metabolism in their hosts.

## Materials and Methods

### Ethical Approval

The animal study was reviewed and approved by the Animal Care Committee of the Institute of Geography and Agroecology, Chinese Academy of Sciences, Jilin, China (Protocol No. 2019003).

### Lambs, Diets, and Experimental Design

#### Third-Stage Infectious Larvae Preparation

Third-stage (*L*3, infectious stage) *H. contortus* larvae were prepared by an egg hatching trial, as described (Hansen and Perry, 1990). In brief, 2 kg of fresh feces were collected from grazing sheep infected with GINs, as confirmed by microscopy. These feces were placed into a hatching basin which was put at ∼25°C and ∼70% relatively humidity to hatch mixed nematode eggs for 15 d. During hatching, the development status of larvae was assessed (light microscopy) based on their shape changes, to determine when most larvae reached the infectious *L*3 stage (oval eggs had just hatched into thread-like nematodes). After hatching, feces were wrapped with four layers of gauze and *L*3 larvae moved from the feces into water. The development status of larval and the species of mixed *L*3 mixed larvae were determined by light microscopy, as described (Hansen and Perry, 1990).

#### Lambs

Sixteen male Ujumqin lambs, 3–4 months old with an average live weight of 32.4 ± 3.9 kg, were reared under helminth-free conditions (verified and confirmed by parasitological examination of individual fecal samples prior to the beginning of the trial). All lambs were dewormed using a combination of abamectin (0.2 mg/kg BW), levamisole (7.5 mg/kg BW), and albendazole (5 mg/kg BW) (Pyrimide^®^, Novaritis Animal Health Co., Ltd., Shanghai, China). After 28 d of deworming, all lambs were randomly allocated into 2 groups (control group, CON and gastrointestinal nematode infection group, GIN), each with eight lambs, and GIN lambs were orally dosed *L*3 *H. contortus*. Before oral dosing of *L*3 *H. contortus*, the worm egg count (WEC) was checked to make sure zero egg burden for all lambs. Infection was induced by drenching each GIN lamb with ∼10,000 mixed *L*3 larvae (89 ± 4% *H. contortus*, 8 ± 4% *Teladorsagia circumcincta* and some other species of nematodes, as assessed by light microscopy (Classica 102M, China, Beijing), which were hatched in one batch before infection. In contrast, CON lambs were orally dosed tap water. After GIN lambs were infected, the formal feeding experiment was started and lasted 77 days.

#### Diets and Management

During the post-infection feeding period, each lamb was housed in a separate pen (140 cm long, 100 cm wide and 124 cm high) with *ad libitum* access to fresh water. All lambs were fed the same diet (Table 1) twice daily at 06:00 and 18:00. At each feeding, according to different feed intake of each lamb during adaption period, each lamb was generally given 700∼900 g concentrate. Then, sufficient corn straw was provided to ensure that each lamb would have no more than ∼10% refusal.

**TABLE 1:** Ingredients and chemical composition of experimental diets.

Item	CON/GIN
**Ingredients (%)**	
Corn straw	40.0
Corn meal	21.0
Soybean meal	10.2
Corn germ meal	9.0
Sunflower seed meal	5.0
Wheat bran	10.5
Limestone	1.0
CaHPO_4_	0.8
NaCl	0.5
Mineral salt and vitamins^a^	2.0
**Chemical composition (n = 8)**	
DM,%	97.8
Crude protein,%	9.3
Ether extract,%	5.4
NDF,%	51.7
ADF,%	24.1
Starch,%	22.6
Metabolizable energy, MJ/kg of DM	13.5

*^a^Mineral salt and vitamins: purchased from Agriportal, were comprised of (per kg): 16.5 g Ca, 8.5 g P, 11.5 g Na, 1.6 g Mg, 1.5 g K, 1.7 g S, 1.25 g Fe, 1.22 g Mn, 1.23 g Z, 240 mg Co, 1,750 mg Cu, 450 mg I, 50 mg Se, 350,000 IU/Ib vitamin A, 55,000 IU/Ib vitamin D3 and 500 IU/Ib vitamin E.*

#### Sample Collection and Analysis Procedures

The daily feed supply and feed refusal for each lamb were recorded to calculate feed intake. Before the morning feeding on days 1, 7, 21, 42, 63, and 77 of the post-infection feeding period, all lambs were weighed and average daily gain (ADG) of each lamb was calculated during the feeding period. A digestibility trial started on day 35 and consisted of 5 days for adaption, followed by 5 days of sampling (total feces) to determine apparent digestibility of feed nutrients. All fresh feces of each lamb from each separate metabolism pen were collected daily (from 06:00 the first day to 06:00 the next day) by picking up each feces particle, sub-sampled (100 g/kg), and stored at −20°C. After 5 d of collection, five subsamples were mixed into a composited sample and stored at −20°C for further analysis. Dietary and fecal samples were dried at 65°C to determine dry matter (DM) and then forced through a 0.425 mm screen to facilitate analysis. Nitrogen content was analyzed (method no. 968.06; AOAC, 1990) and crude protein (CP) content was calculated as 6.25 × nitrogen content. The Van Soest detergent fiber analysis method was used to determine neutral detergent fiber (NDF) and acid detergent fiber (ADF) (Van Soest et al., 1991). Ash content was measured using the methods of the Association of Official Analytical Chemists (method 924.05; AOAC, 1990) and ether extract (EE) content was measured as described (AOAC, 1995).

On days 1, 3, 7, 14, 28, 42, 56, 63, and 77 post-infection, WEC was determined by the modified McMaster’s technique (Whitlock, 1948) and expressed as eggs per gram (EPG) of feces. Briefly, 2 g of crushed feces which was collected from the rectum was mixed with 28 mL of saturated saline and this solution was aspirated into the McMaster’s egg counting plate, the WEC was determined through a light microscope (Classica 102M, China, Beijing) in lab. At last, EPG was calculated from WEC (EPG = FEC × 50).

On days 1, 3, 7, 14, 21, 42, 63, and 77 post-infection, 7 mL of blood was collected from the jugular vein of each lamb, with 2 mL put into a sterile tube containing EDTA-K2 and PCV determined with a blood cell analyzer (Sysmex XE-2100, Shanghai, China). The remaining 5 mL of blood was put into a tube containing heparin sodium and the tubes were centrifuged at 2,500 × g for 15 min. Plasma free amino acid concentrations were determined as described (Jiao et al., 2020), using an automatic amino acid analyzer (Hitachi L-8900, Tokyo, Japan).

At 77 d after infection, all lambs fasted for 12 h, electrically stunned, and exsanguinated under commercial conditions. The current was delivered at a constant voltage of 220 V, 1.0 A for 3 s with scissor tongs applied using a head-only stunner, with electrodes between the eyes and ears on either side of the head. Lambs were exsanguinated within 20 s after stunning. Chyme in the rumen, abomasum, duodenum, jejunum, ileum and colon of each lamb was collected and immediately stored at −80°C for microbiome sequencing and assessment of gastrointestinal digestive enzyme activity.

Microbiome sequencing was done as described (El-Ashram et al., 2017). The ruminal, abomasal and duodenal chyme were centrifuged at 5,000 rpm for 5 min, and the supernatant was centrifuged at 12,000 rpm for 10 min. Total genomic DNA of the microbiota was extracted from each chyme sample using the QIAamp Fast DNA Stool Mini Kit (Qiagen, Hilden, Germany) according to manufacturers’ instructions. DNA concentration was quantified using a QuantiFluor fluorometer (Promega, United States). The region of bacterial 16S rRNA was used to characterize microbial populations in chyme samples. Universal primers were used to amplify the V3 and V4 hypervariable regions of the 16S rRNA gene library. The primers were 341F (5′-CCTAYGGGRBGCASCAG-3′) and 806R (5′-GGACTACNNGGGTATCTAAT-3′) (Lin et al., 2019). The PCRs were performed as follows: initial denaturation was 95°C for 5 min, followed by 30 cycles of 98°C for 10 s, 50°C for 30 s and 72°C for 30 s, with a final extension step at 72°C for 5 min. Pooled amplification products were purified with QIAauick Gel extraction Kit (Qiagen) according to manufacturer’s instructions. Subsequently, the amplicon library was prepared with TruSeq^®^ DNA PCR-Free Sample Preparation Kit (Illumina, United States) and quantified with QuantiFluor™-ST (Promega, United States). The library pool was sequenced using an Illumina MiSeq Reagent Kit on an Illumina MiSeq sequencer, with NovaSeq6000 used for on-board sequencing. Pepsin, chymotrypsin, trypsin, cellulase, lipase and α-amylase activity were measured with a UV spectrophotometer (Shimadzu UV-1,800, Beijing, China), using commercial kits (Jianchen, Nanjing, China).

### Statistical Analyses

Data for feed nutrient apparent digestibility and digestive enzyme activity were analyzed using a General Linear Model, followed by Duncan’s multiple range tests (SAS, 2002). Data for PCV, EPG and free amino acid profiles were analyzed with a MIXED model, as described (Littell et al., 1996). The model consisted of treatments, post-infection days, treatments × post-infection days interactions as fixed effects, and animals as the random effect. Measurements obtained from each lamb on various sampling days were treated as repeated measures. Means were separated using the least squares mean and presented with the standard error of the mean. Differences were considered significant if *P* ≤ 0.05. Figures were made with OriginPro 2021.

QIIME pipeline (Version 1.9.1) was used to calculate indexes of Observed OTUs, Chao1, Shannon, Simpson, ACE, Goods-coverage and PD whole tree. The software R (Version 2.15.3) was used to draw the rarefaction curve and analyze the statistical significance of Alpha diversity index by Wilcoxon rank sum test. The OTU relative abundance values were analyzed using the LEfSe algorithm to identify taxa with significant differences between treatments. Furthermore, Tax4Fun was used to predict gene contents and metagenomic functional information based on SILVA SSU Ref NR database. Differences were considered significant when *P* < 0.05.

## Results

### Packed Cell Volume, Dry Matter Intake, Average Daily Gai, and Digestibility

**FIGURE 1:**
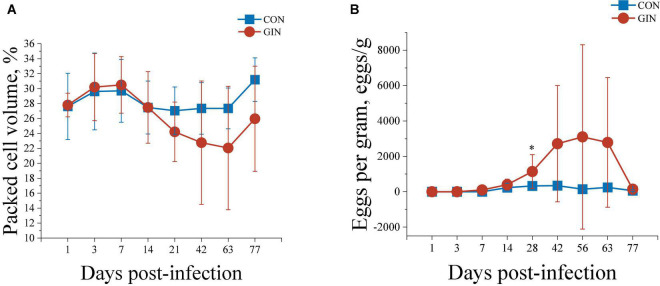
The PCV **(A)** and WEC **(B)** of lambs infected with Haemonchus contortus.*P < 0.05.

Infection with *H. contortus* decreased packed cell volume (PCV) and increased EPG. Briefly, the PCV in GIN was lower than in CON lambs during 14∼77 days post-infection (*P* < 0.05) (Figure 1A). Furthermore, EPG of GIN lambs increased after 28 d infection (*P* < 0.05) and remained higher than in CON lambs during 28∼63 days post-infection, although not significantly different. Finally, the EPG of GIN lambs reduced to a similar level with CON lambs on day 77 (*P* > 0.05) (Figure 1B).

*H. contortus* infection decreased dry matter intake (DMI) of lambs by 20.7% (*P* < 0.001), reduced average daily gain (ADG) (*P* < 0.001) (Table 2) and decreased the apparent digestibility of dietary DM (*P* = 0.011), CP (*P* = 0.004), and EE (*P* = 0.007), but did not change the apparent digestibility of dietary NDF, ADF, or ash (*P* > 0.05).

**TABLE 2:** Growth performance and dietary nutrient apparent digestibility of lambs infected with *Haemonchus contortus*^a^.

Item^b^	Treatment		SEM	*P*-value
	CON	GIN		
DMI (g/d)	1,294	1,026	43.39	<0.001
Initial weight (kg)	13.24	13.46	0.803	0.548
Final weight (kg)	15.85	13.11	1.586	0.001
ADG (g/d)	110.4	−14.45	16.76	<0.001
**Apparent digestibility,%**				
DM	67.50	64.33	0.683	0.011
CP	63.99	55.46	1.507	0.004
NDF	62.77	59.85	1.922	0.315
ADF	36.18	27.86	2.667	0.059
Ether extract	77.80	70.76	1.397	0.007
Ash	87.48	55.46	2.696	0.968

*^a^Values are presented as means ± SEM and significance between different treatments was assessed by a P-value of less than 0.05. The same as below.*

*^b^DMI, dry matter intake; ADG, average daily gain; DM, dry matter; CP, crude protein; NDF, neutral detergent fiber; ADF, acid detergent fiber.*

### Digestive Enzyme Activities

For protein-degrading enzymes, *H. contortus* infection decreased activity of ruminal pepsin (*P* < 0.001) and ileac trypsin (*P* = 0.025) but did not affect chymotrypsin activity in any part of the gut (Table 3). For starch digestion, *H. contortus* infection increased activity of ruminal α-amylase (*P* = 0.004) but decreased its activity in the jejunum (*P* = 0.033). For lipid and fiber digestion, *H. contortus* infection decreased ruminal (*P* = 0.032) and duodenal (*P* = 0.040) lipase activity, increased ruminal cellulase activity (*P* = 0.002), but did not affect their activity in other parts of the gastrointestinal tract (*P* > 0.05).

**TABLE 3:** Activity of gastrointestinal digestive enzymes of lambs infected with *Haemonchus contortus*.

Item		Treatment		SEM	*P-*value
		CON	GIN		
Pepsin (U/mg)	Rumen	13.59	4.300	1.129	<0.001
	Abomasum	134.9	119.8	6.876	0.151
Trypsin (U/mg)	Duodenum	2,798	3,080	490.5	0.692
	Jejunum	3,500	2,755	300.6	0.110
	Ileum	320.1	107.0	56.99	0.025
	Colon	1,260	1,242	566.5	0.986
Chymotrypsin(U/mg)	Duodenum	3.300	3.750	1.306	0.814
	Jejunum	0.710	1.440	0.510	0.333
	Ileum	2.000	1.670	0.893	0.799
	Colon	3.200	2.690	0.477	0.469
α-amylase (U/mg)	Rumen	0.480	1.290	0.152	0.004
	Abomasum	2.910	1.560	0.967	0.349
	Duodenum	2.670	3.000	0.428	0.591
	Jejunum	3.160	2.100	0.302	0.033
	Ileum	1.300	3.440	1.005	0.163
	Colon	0.920	1.860	0.336	0.078
Lipase (U/mg)	Rumen	120.0	75.61	12.61	0.032
	Abomasum	86.71	73.08	16.99	0.583
	Duodenum	196.2	53.60	42.68	0.040
	Jejunum	31.39	39.90	5.506	0.300
	Ileum	15.52	30.88	6.674	0.257
	Colon	37.79	32.68	3.361	0.308
Cellulase (U/mg)	Rumen	391.1	1,307	158.9	0.002
	Abomasum	685.5	435.0	137.3	0.226
	Duodenum	344.1	338.0	70.99	0.953
	Jejunum	169.7	171.1	40.88	0.981
	Ileum	854.7	672.5	205.8	0.545
	Colon	1,465	1,008	248.2	0.223

### Amino Acid Profiles

**FIGURE 2:**
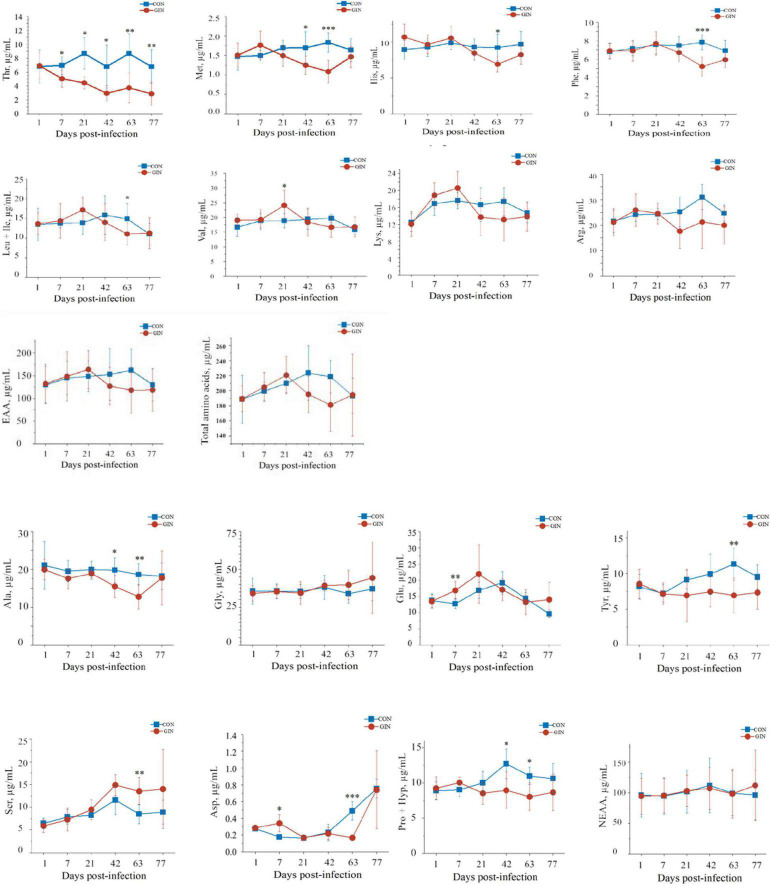
Changes in the plasma free amino acids profile of lambs infected with *Haemonchus contortus.* *P < 0.05; ^**^P < 0.01; ^***^P < 0.001.

*H. contortus* infection altered plasma amino acid profiles and for most amino acids, their concentrations were significantly different between treatments on days 7, 42, and 63 post-infection (Figure 2). Plasma concentrations of essential amino acids (EAA) decreased after 42 days post-infection but did not reach significance (*P* > 0.05). Similarly, there was no difference between CON and GIN (*P* > 0.05) in concentrations of non-essential amino acids (NEAA). However, concentrations of aspartic acid (Asp) (*P* = 0.003) and glutamine acid (Glu) (*P* = 0.008) in GIN were higher than those in CON on day 7. Concentrations of Met (*P* = 0.045), alanine (Ala) (*P* = 0.036) and conditional EAA (proline and hydroxyproline) (CEAA, Pro + Hyp, *P* = 0.020) were lower in GIN than in CON on day 42. On day 63, concentrations were lower in GIN than in CON for the following: Met (*P* = 0.001), phenylalanine (Phe) (*P* = 0.001), threonine (Thr) (*P* = 0.007), leucine (+ isoleucine) (Leu + Ile)(*P* = 0.017), histidine (His) (*P* = 0.024), tyrosine (Tyr) (*P* = 0.008), Ala (*P* = 0.008), Asp (*P* < 0.001), serine (Ser) (*P* = 0.007), EAA (*P* = 0.011) and CEAA (Pro + Hyp) (*P* = 0.011); however, there were no differences for concentrations of valine (Val), lysine (Lys), arginine (Arg), glycine (Gly), Glu, and NEAA (*P* > 0.05). In addition, plasma Val concentrations were higher in GIN than CON on day 21. Notably, plasma Thr concentrations in GIN were lower than in CON from days 7 to 77 (*P* < 0.05).

### Microbiota Profile

**FIGURE 3:**
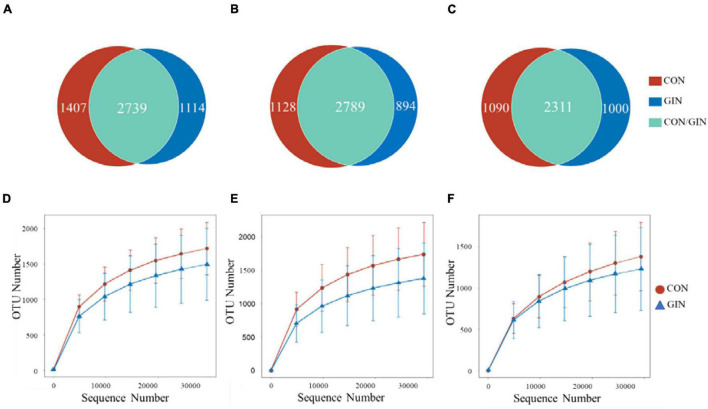
Venn diagrams of rumen, abomasum and duodenum is **(A–C)**, respectively and rarefaction curves of rumen, abomasum and duodenum is **(D–F)** of lambs infected with *Haemonchus contortus*.

A total of 529,532/579,319/584,093 high-quality reads were obtained from the ruminal/abomasal/duodenal chyme samples which were collected from 12 lambs, with a mean of 44,128/48,277/48,674 reads per sample from the rumen, abomasum and duodenum, respectively. The number of OTUs detected in each ruminal sample ranged from 838 to 2,207 with a mean of 1,587; the number of OTUs detected in each abomasal sample ranged from 546 to 2,286 with a mean of 1,540; and the number of OTUs detected in each duodenal sample ranged from 574 to 1,915 with a mean of 1,296. The OTUs in the rumen, abomasum and duodenum of CON/GIN were 1950.67 ± 569.62/1559.33 ± 623.16, 1920.50 ± 488.85/1651.00 ± 651.83, and 1617.50 ± 519.03/1392.50 ± 651.36, respectively. There were 2,739, 2,789, and 2,311 shared OTUs in the rumen, abomasum and duodenum of both groups, respectively, and 33.94/28.91, 28.80/24.27, and 32.05%/30.20% unique OTUs in the rumen, abomasum and duodenum of CON/GIN, respectively (Figure 3). Rarefaction curves generated for the OTU number nearly reached asymptotes at read depths of 30,000 for all samples, indicating the sampling depth provided sufficient coverage for comprehensive analysis of bacterial composition. Furthermore, good’s coverage was > 0.97 for all samples, implying that depth of coverage met requirements of subsequent measurements.

Based on rarefaction curves, species richness of ruminal, abomasal, and duodenal chyme were lower in GIN vs. CON (Figure 3). However, based on a comparison between treatments of observed species values of chyme in abomasum (Wilcoxon rank sum test: *P* = 0.220), rumen (*P* = 0.452) and duodenum (*P* = 0.615), species richness reduction was not obvious by GIN infection. Similarly, there were no significant differences between treatments in Chao1, Shannon, good coverage and PD whole tree indexes of ruminal, abomasal and duodenal chyme, and the Simpson indices of ruminal and duodenal chyme were not affected by *H. contortus* infection (*P* > 0.05). By contrast, for abomasal chyme, the Simpson value differed between treatments (*P* = 0.034) (Table 4).

**TABLE 4:** Alpha diversity index of microbiota in rumen, abomasum and duodenum of lambs infected with *Haemonchus contortus*.

Index	Rumen			Abomasum			Duodenum		
	CON	GIN	*P-*value	CON	GIN	*P-*value	CON	GIN	*P*-value
Observed species	1,709	1,463	0.451	1,722	1,356	0.220	1,374	1,217	0.615
Good’s coverage	0.987	0.989	0.414	0.988	0.989	0.686	0.985	0.989	0.303
Shannon	8.189	7.088	0.109	8.219	7.055	0.052	6.065	6.427	0.661
Chao 1	1,958	1,651	0.378	1,921	1,599	0.378	1,800	1,434	0.307
Simpson	0.987	0.921	0.103	0.983	0.961	0.034	0.911	0.942	0.632

**FIGURE 4:**
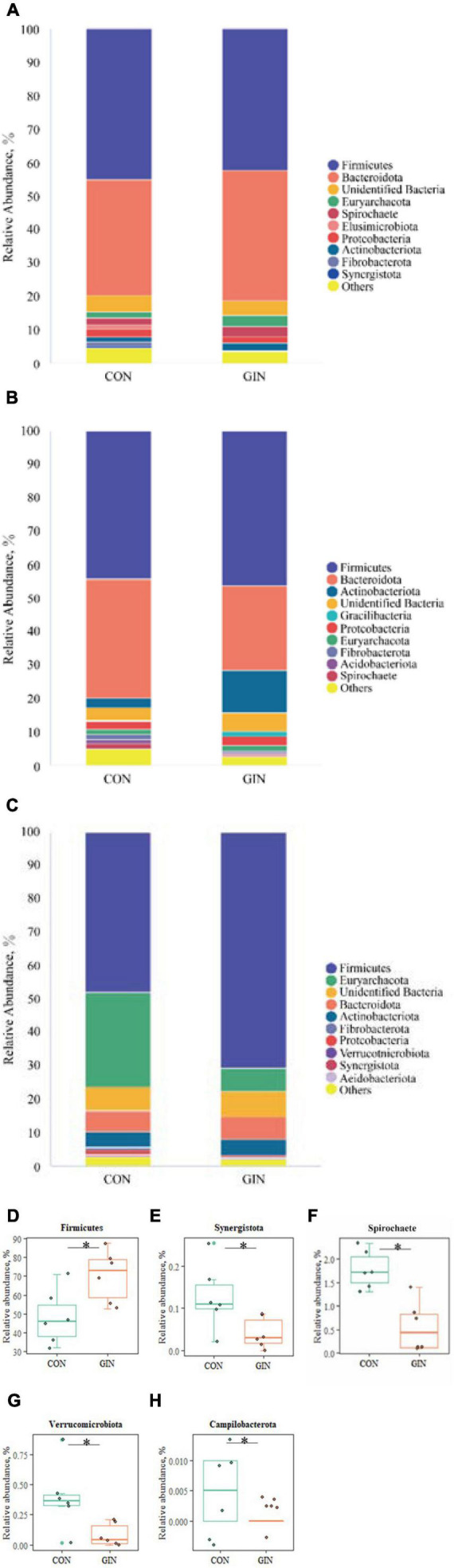
The top 10 phyla and other phyla of the rumen, abomasum and duodenum of lambs infected with *Haemonchus contortus.* **(A–C)** The top 10 phyla in the rumen, abomasum and duodenum; **(D)** significantly different phyla in the duodenum; **(E–H)** significantly different phyla in abomasum. *P < 0.05.

A diverse range of bacterial phyla were identified in all chyme samples, including Firmicutes, Bacteroidota, Euryarchaeota, Actinobacteriota, Proteobacteria, and Spirochete (Figure 4). Relative proportions of these phyla differed between treatments (*P* < 0.05) in the abomasum and duodenum, but not in the rumen. In the GIN treatment, abomasal Spirochete (*P* = 0.013), Verrucomicrobiota (*P* = 0.025), Campilobacterota (*P* = 0.028) and Synergistota (*P* = 0.041) were lower, whereas in the duodenum, Firmicutes was higher (*P* = 0.041).

**FIGURE 5:**
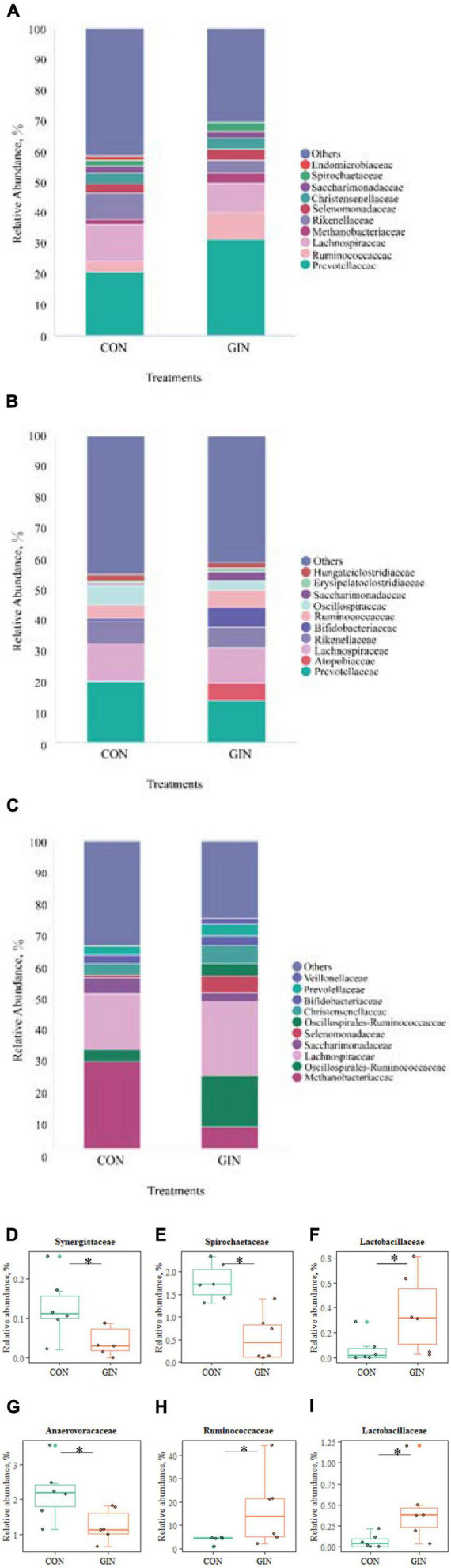
The top 10 families and significantly different families of rumen, abomasum and duodenum. **(A–C)** The top 10 families of rumen, abomasum and duodenum; **(D–F)** significantly different families in abomasum; **(G–I)** significantly different families in duodenum. *P < 0.05.

There was a clear shift in microbial proportions at the family level, with specific families of taxa increasing or decreasing in abundance during the course of infection (Figure 5). *H. contortus* infection changed a variety of abomasal microbial families, with an increase in abomasal Lactobacillaceae (*P* = 0.026) and decrease in Spirochaetaceae (*P* = 0.005) and Synergistaceae (*P* = 0.041). In addition, in the GIN treatment, the family of duodenal Lactobacillaceae (*P* = 0.030) and Ruminococcaceae (*P* = 0.041) increased, whereas duodenal Anaerofustaceae decreased (*P* = 0.041).

**FIGURE 6:**
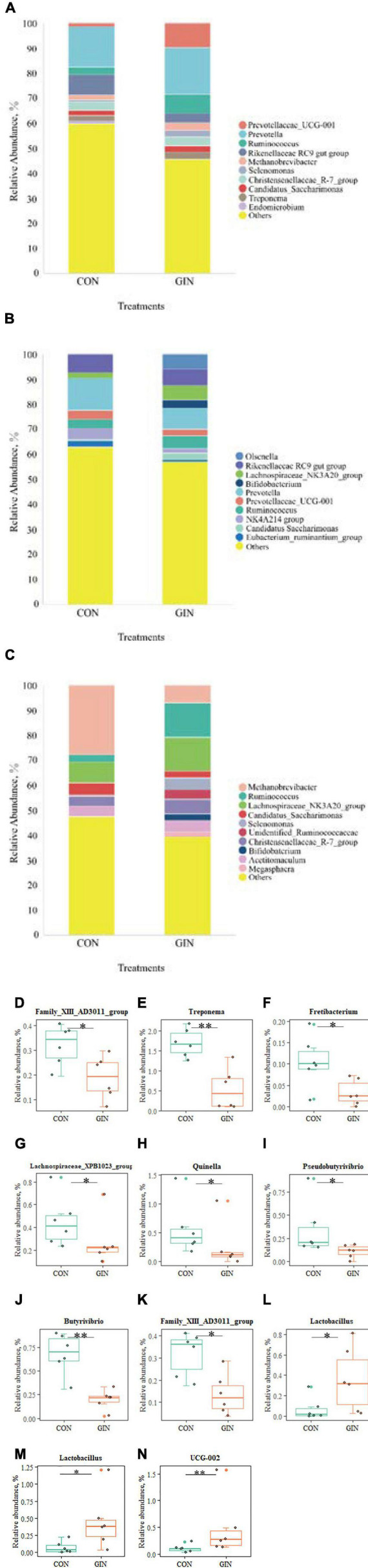
The top 10 genus and significant different genus of rumen, abomasum and duodenum. **(A–C)** The top 10 genus of rumen, abomasum and duodenum; **(D)** significantly different genus in rumen; **(E–L)** significantly different genus in abomasum; **(M,N)** significantly different genus in duodenum. *P < 0.05, ^**^P < 0.01.

Microbial community composition at the genus level also differed between treatments (Figure 6). In the GIN treatment, the genus of *Family XIII AD3011 group* in rumen decreased (*P* = 0.048), but there was increased *UCG-002* (*P* = 0.009) and *Lactobacillus* (*P* = 0.030) in duodenal chyme. *H. contortus* infection caused a variety of genus changes in the abomasal chyme microbial community, with declines in *Treponema*, *Fretibacterium*, *Lachnospiraceae XPB1014 group*, *Quinella*, *Family XIII AD3011 group*, *Butyrivibrio* and *Pseudobutyrivibrio* (*P* < 0.05). However, the genus *Lactobacillus* increased (*P* = 0.026).

**FIGURE 7:**
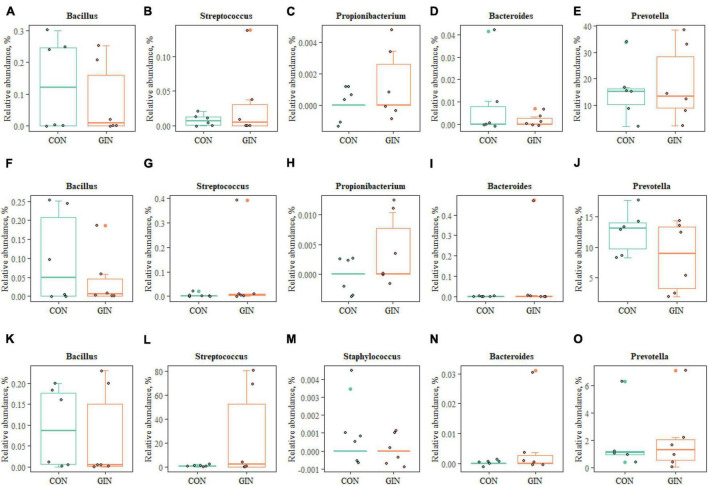
The proteolytic bacteria of rumen **(A–E)**, abomasum **(F–J)** and duodenum **(K–O)** at genus level.

Regarding proteolytic bacteria in duodenal chyme, samples in the GIN treatment also differed from those in the CON treatment at the genus level (Figure 7). In the GIN treatment, there were significant increases in: the genus of *Prevotella*, *Propionibacterium*, and *Streptococcus* in ruminal chyme; the genus of *Bacteroides*, *Propionibacterium*, and *Streptococcus* in abomasal chyme; and the genus of *Prevotella*, *Bacteroides*, and *Streptococcus* in duodenal chyme.

The KEGG pathways of duodenum microbiota are shown (Table 5). It is noteworthy that some pathways closely related to metabolism of amino acids, carbohydrate and lipids were changed by *H. contortus* infection (*P* < 0.05).

**TABLE 5:** KEGG pathways of the duodenum microbiota significantly affected by *Haemonchus contortus* infection in lambs.

KEGG pathway		Treatment		*P* Value
		GIN	CON	
Amino acids metabolism	Nitrogen metabolism	0.595	0.508	<0.001
	beta Alanine metabolism	0.154	0.111	<0.001
	D-Glutamine and D-glutamate metabolism	0.130	0.136	0.003
	Taurine and hypotaurine metabolism	0.108	0.076	0.006
	Phosphonate and phosphinate metabolism	0.037	0.020	0.005
	Selenocompound metabolism	0.415	0.392	0.021
	D-Alanine metabolism	0.086	0.054	0.054
	Cyanoamino acid metabolism	0.210	0.170	0.130
	D-Arginine and D-ornithine metabolism	0.002	0.001	0.262
	Glutathione metabolism	0.142	0.126	0.408
Carbohydrate metabolism	Fructose and mannose metabolism	0.502	0.405	0.001
	Pentose phosphate pathway	0.651	0.604	0.004
	Galactose metabolism	0.672	0.510	0.010
	Pentose and glucuronate interconversions	0.298	0.231	0.022
	Various types of N-glycan biosynthesis	0.051	0.101	0.049
	Amino sugar and nucleotide sugar metabolism	1.209	1.148	0.037
Lipid metabolism	Primary bile acid biosynthesis	0.018	0.023	0.027
	Secondary bile acid biosynthesis	0.018	0.023	0.011
	Arachidonic acid metabolism	0.016	0.010	0.019
	Glycerolipid metabolism	0.267	0.160	0.023
	Biosynthesis of unsaturated fatty acids	0.090	0.063	0.024
	Sphingolipid metabolism	0.185	0.107	0.033

## Discussion

### Effects of *Haemonchus contortus* Infection on Packed Cell Volume, Growth and Digestion

The WEC and PCV are preferred parameters to indicate GIN infection status. Our present results were consistent with previous reports that *H. contortus* infections consistently increase WEC and decrease PCV due to their blood sucking (Khan et al., 2017; Silva et al., 2019). The reason for the WEC to go back to zero at 77 days may be connected with cold temperature (Lucius et al., 2017). Furthermore, DMI and ADG of GIN lambs decreased by 20.7 and 113.1%, respectively, when compared to CON lambs, consistent with reports that *H. contortus* infections reduce growth performance of host ruminants, attributed to the infection decreasing feed intake, resulting in anorexia (Jones et al., 2006) and suppressing digestion and absorption of dietary nutrients (Angulo-Cubillan et al., 2007).

In the present study, dietary protein and lipid metabolism were altered by the *H. contortus* infection. Many studies concluded that GIN infection had negative effects on feed intake and nutrient digestibility (Coop and Holmes, 1996; Coop and Kyriazakis, 1999; Cardia et al., 2011). Meanwhile, GIN infection in sheep has been reported to impair rumen function and metabolism, increase the pH of rumen and decrease the degradability of the feed (Doyle et al., 2011). Furthermore, Bown et al. (1991) reported that GIN (*T. colubriformis* and *O. circumcincta*) infection alters metabolism of protein more than other components. In addition, based on higher α-amylase activity, *H. contortus* infection increased carbohydrate metabolism in the rumen, compensating for the loss of energy supply from depressed protein and lipid metabolism and a potential reason why protein metabolism was largely affected by infection rather than fiber metabolism (Houdijk et al., 2012). Rumen function of infected lambs may be altered in response to *H. contortus* infection. Doyle et al. (2011) showed that sheep infected with *H. contortus* had an increase in fluid outflow, turnover rate, and a decrease in propionic acid concentration which may be the reason why sheep develop resistance to *H. contortus* infection.

### Amino Acid Profiles

Plasma amino acid profiles were measured to elucidate effects of *H. contortus* infection on host protein metabolism. Some amino acids, especially some EAA, were preferentially used for regulating immune responses, tissue repair, and to support *H. contortus* growth which reflects that the redistribution of amino acid resources may related with the development of GIN (Coop and Holmes, 1996; Colditz, 2010; Doyle et al., 2014).

In the present study, plasma Thr concentrations were markedly decreased in infected lambs. *H. contortus* infections damage the host’s gastrointestinal mucosa, causing enteritis (Hoste et al., 2006). Furthermore, Thr is abundant in the mucin that lines the gut (Zhang et al., 2019). Expression of protein regulated genes related to mucin secretion in the gastrointestinal tract of animals decreased when dietary Thr was deficient (Emadinia et al., 2020). Besides, Thr has an important role in preventing the bite of nematodes and inhibiting the colonization of nematodes in parasitic habitats (Emadinia et al., 2020). Perhaps decreased plasma Thr concentrations were due to synthesizing mucin repairing gastrointestinal damage. Furthermore, there was an inverse relationship between plasma Thr concentrations and WEC, so the former may be an indicator to judge the severity of *H. contortus* infection in small ruminants.

WEC peaking on day 42 post-infection indicated that the parasitic larvae had developed into adults and reached peak spawning. Concurrently, there were decreases in plasma Pro and Arg concentrations, two amino acids required for nematode establishment, reproduction, and survival. Nematode eggs are composed of proline-rich proteins and each egg is enclosed by a stratum corneum, forming a barrier between itself and the host’s gastrointestinal environment (Page et al., 2014). In addition, Pro is also involved in regulation of nematode osmotic pressure (Barrett, 1991). Consequently, adult nematodes derive a large amount of Pro from host blood to support growth and reproduction (Page et al., 2014). In addition, Arg can promote attachment and growth of GINs and act as a nematode-secreted protein interaction factor to initiate the host’s non-specific defense mechanisms (James, 1995).

In the present study, severe shedding of wool occurred in most GIN lambs. Wool protein has a relatively high requirement for Met and Cys, the sulfur amino acids (Adams and Liu, 2003). Furthermore, the irreversible loss rate (an index reflecting effects of parasitic infection on protein turnover) of Met and Cys, was reduced by 13∼15% in GIN lambs (Bermingham, 2000; Liu et al., 2002). Therefore, changes in plasma Met and Cys concentrations were consistent with wool loss in GIN lambs. Furthermore, Met and Cys are involved in activation of immune responses. In that regard, Cys is the substrate of some immune proteins and inflammatory factors, e.g., glutathione (GSH) (Grimble, 2006).

Changes in plasma EAA concentrations appeared to be associated with immune functions during parasitic infections (Houdijk et al., 2012). In addition to Met and Cys, there is increased utilization of His, Phe and Asp in production of proteinaceous immune response components, enhancing host immune responses and promoting growth of intestinal mucosa (Liu et al., 2008; Zhang et al., 2013). Increased plasma Asp concentration on day 7 post-infection in the present study was in contrast to the decrease at day 63; and perhaps its function changes in different stages of *H. contortus* infection. We inferred that increased Asp on day 7 was due to its primary role for synthesis of Thr, which is in high demand during infection, as noted above (Emadinia et al., 2020). There are reports of similar changes in utilization of amino acids of animals infected by GINs. For example, Leu utilization differed at 18∼20 week of GIN infection compared to 5∼7 or 11∼13 week (Yu et al., 2000). In addition to Asp, plasma concentrations of Glu, Ser and Tyr increased during *H. contortus* infection. It is noteworthy that Glu cannot be synthesized by the host and has an important role in maintaining host nitrogen balance and biosynthesis of Gln, Arg and Pro. In addition, Glu contributes to energy generation and production of host gastrointestinal tract mucosa (Reeds, 2000). Consistent with the findings in lambs infected with *T. colubriformis* (Roy et al., 2003), the current results of increased plasma Glu and Asp content from days 1 to 21 indicated that the host might mobilize more Glu to be absorbed into the blood to prevent against parasitic larvae colonization. Therefore, the urgent need of Asp and Glu seem to be an important marker of invasion by *H. contortus*.

Increased Ser may be associated with nutrient transfers caused by *H. contortus* infection. Infections with GIN infection can trigger the transfer of nutrients from production sites to other tissues to synthesize key proteins (Cardia et al., 2011), causing significant increases in concentrations of amino acids (e.g., Ser) related to skeletal muscle formation (Rowe et al., 1978). Decreased plasma Tyr concentrations may be due to Tyr being used to synthesize Phe.

In summary, *H. contortus* infection increased protein requirements for maintenance, repair of parasite-induced damaged tissue, production of proteinaceous immune response components, for nematode establishment, fecundity and survival. Therefore, plasma amino acid concentrations changed with increased requirements to meet demands.

### Microbiota Composition

GIN infections may change the composition of the host’s gastrointestinal microbial community (Li et al., 2016; Wang et al., 2019). Alpha diversity is defined as the average species diversity within a microbial population (Tuomisto, 2010). Increased alpha diversity in the gastrointestinal microbiota is usually related to healthy gastrointestinal homeostasis. In contrast, many inflammatory responses, e.g., gastrointestinal diseases and GIN infection, decrease alpha diversity (Bak et al., 2015; Cattadori et al., 2016). Similarly, in the present study, microbiota associated with host health decreased, including the genus of *Lachnospiraceae XPB1023 group*, *Treponema*, *Butyrivibri*, and *Pseudobutyrivibrio* (the so-called butyrate-producer bacteria) in the abomasum. Butyrate, with its involvement in the energy supply and gastrointestinal tract epithelial health, is a major microbial fermentation product in gastrointestinal tract. Our results were in accordance with reports of a lower abundance of butyrate-producing bacteria in goats infected by *H. contortus* (Li et al., 2016) and *Cryptosporidium parvum* (Mohamed et al., 2020). However, in some studies, alpha diversity did not change or increase during GIN infection (Li et al., 2012; McKenney et al., 2015). These apparent differences may be due to different animal or parasite species, as well as sampling times. Acute inflammatory episodes after parasite invasion of the gastrointestinal tract are likely accompanied by decreased microbial diversity, which may subsequently be restored.

Despite decreased microbiome diversity in the rumen, abomasum, and duodenum, the abundance of some key microbiota increased in the GIN group. These increased microbiotas may restore nutrient metabolism in the host lambs. There was an increase in microbiota associated with cellulose breakdown, e.g., phylum of Firmicutes (including the genus of *UCG-002*) in duodenum and the family of Ruminococcoceae. The latter, and Firmicutes, degrade cellulose and hemicellulose (Jennifer et al., 2009; Biddle et al., 2013), is consistent with the absence of a decrease in NDF and ADF apparent digestibility in the GIN treatment. Higher abundance of fiber-degrading microbiota could have compensated for the disruption of digestion and absorption caused by mucosal damage.

Fermentability and physical properties of host dietary carbohydrates affect establishment, distribution and fecundity of GINs (Petkevicius et al., 2007). In the present study, some microbiotas associated with starch and other carbohydrates metabolism increased, including the family of Lactobacillaceae, the genus of *Lactobacillus* (and the species of *Lactobacillus amylovorus*, *Lactobacillus reuteri*) and *Treponema* appeared in the abomasum and duodenum rather than the rumen. Furthermore, *L. amylovorus* and *L. reuteri* are the most abundant species among *Lactobacillus* spp. (Splichal et al., 2019). *Lactobacillus* are called probiotics; *in vitro*, *Lactobacillus* released antimicrobial substances (Foster et al., 2003; Tierney et al., 2004), and end products of carbohydrate fermentation, including short-chain fatty acids (such as butyrate and acetate) which can enhance functions of epithelial cells. Adding probiotics increased glucose absorption in pig small intestines during *Ascaris suum* infection, which can in turn promote expelling of GIN (Li et al., 2012). So, increased *Lactobacillus* has a therapeutic role. Furthermore, increased *Lactobacillus* and *Treponema* are non-pathogenic, carbohydrate-metabolizing bacteria that can enhance the supply of nutrients needed in response to GIN infection (Flint et al., 2012; Allison et al., 2018; Zúiga et al., 2020). In addition, KEGG pathways related to carbohydrate metabolism were enhanced in infected lambs. Therefore, we inferred those compensatory signatures were induced to degrade carbohydrates to supply nutrients and energy needed by hosts.

Carbohydrate metabolism is related to protein metabolism, as common metabolic intermediates from protein metabolism are also involved in carbohydrate metabolism, e.g., tricarboxylic acid cycle, pentose phosphate pathway and glycolysis (Mu et al., 2015). In theory, carbohydrate metabolism promotes uptake and utilization of nitrogen-containing substances by hosts. Infected animals have increased gastrointestinal protein leakage and increased shedding of epithelial tissues, resulting in increased gastrointestinal protein losses and requirements (Poppi et al., 1986; Bown et al., 1991). However, the host has compensatory abilities to reabsorb and utilize nutrients, e.g., increased rumen protein digestion and/or reabsorption and utilization of rumen by-pass protein in the duodenum when protein digestion in the abomasum is suppressed due to parasite infection (Poppi et al., 1986). Furthermore, we inferred that microbiota composition may also have an important role in compensating for protein metabolism of lambs infected by *H. contortus*. In the present study, microbiota associated with protein metabolism increased in the GIN treatment. *Prevotella* (the most important proteolytic bacteria for ruminants) increased in the rumen and duodenum, but decreased in the abomasum, opposite to the results of Li et al. (2016). The genus of *Bacteroides*, *Propionibacterium*, *Clostridium*, *Streptococcus*, *Staphylococcus*, *Bacillus*, *Prevotella*, and the family of Ruminococcaceae are the main proteolytic bacteria (Mafra et al., 2013; Wu et al., 2020; Zhao et al., 2021). Increases in these bacteria may improve reabsorbtion and utilization of protein and amino acids and thus influence production of key metabolites. In addition, KEGG pathways of amino acid metabolism were increased in the GIN treatment. Therefore, although *H. contortus* in the abomasum reduced protein uptake, there was increased protein utilization in the host gastrointestinal tract to address requirements.

In addition, there was also an association between protein/amino acid metabolism and changes in composition of gastrointestinal microbiota. Whereas the host provides nutrients to gastrointestinal microbiota and nutrient deficiencies will cause microbial changes, microbiota also produce amino acids that can be used by the host and compensate for amino acid deficiencies caused by infection. There are variations among gut bacteria synthesis and metabolism of amino acids and proteins. For instance, *Clostridium perfringens* lacks genes for biosynthesis of various amino acids (e.g., Glu, Arg, His, Lys, Met, Ser, Thr as well as aromatic and branched-chain amino acids) (Portune et al., 2016), whereas other *Clostridium* species such as *Clostridium acetobutylicum* have a complete set of genes for amino acid biosynthesis (Nölling et al., 2001). Therefore, we inferred that the microbiota composition changes in the present results were closely related to changes in plasma amino acid concentrations and biosynthesis of amino acids by gastrointestinal microbiota. This may regulate amino acids homeostasis in a host infected with nematodes, although regulation mechanisms of amino acid homeostasis by the microbiota in the host has not been well characterized.

## Conclusion

In our study, *H. contoutus* infection decreased feed intake, inhibited apparent digestibility (especially of dietary protein and lipids) and reduced growth performance. In addition, protein absorption was disturbed, plasma amino acid profiles were altered, and ruminal pepsin and ileac trypsin activity decreased. However, infected lambs redistributed absorbed amino acids, with decreased plasma concentrations of Trp, Pro and Arg and increased plasma concentrations of Asp, Glu, Ser and Tyr, to compensate for protein and amino acids losses due to *H. contortus* colonization, development and reproduction. These physiological and metabolic changes in *H. contortus* infection lambs were closely associated with changes in gastrointestinal microbial community composition. The most important finding was an increase in carbohydrate and proteolytic bacteria genus. The present study provided new insights into physiological consequences of *H. contortus* infection in ruminants, which not only improved ruminant gastrointestinal health regulation theories and technologies, but also facilitated establishment of novel indicators, such as plasma amino acid profile, to evaluate or diagnose infection status in small ruminants.

## Data Availability Statement

The original contributions presented in the study are publicly available. This data can be found here: NCBI: USB10549205.

## Ethics Statement

The animal study was reviewed and approved by the Animal Care Committee of the Institute of Geography and Agroecology, Chinese Academy of Sciences, Jilin, China (Protocol No. 2019003).

## Author Contributions

HX: investigation, methodology, software, data curation, and writing-original draft. YF: supervision, validation, writing-review, and editing. ZT: conceptualization, supervision, writing-review, and editing. RZ: conceptualization, methodology, software, data curation, supervision, validation, writing-review, and editing, funding acquisting. All authors contributed to the article and approved the submitted version.

## Conflict of Interest

The authors declare that the research was conducted in the absence of any commercial or financial relationships that could be construed as a potential conflict of interest.

## Publisher’s Note

All claims expressed in this article are solely those of the authors and do not necessarily represent those of their affiliated organizations, or those of the publisher, the editors and the reviewers. Any product that may be evaluated in this article, or claim that may be made by its manufacturer, is not guaranteed or endorsed by the publisher.
